# Azvudine for COVID-19 in Kidney Transplant Recipients: A Real-World Observational Study of Long-Term Renal Outcomes

**DOI:** 10.3390/jcm15145417

**Published:** 2026-07-10

**Authors:** Xiaoyu Li, Xin Xu, Wenyuan Leng, Shufang Deng, Zhenpeng Zhu, Meng Zhang, Wenke Han, Yaqun Zhang, Gengyan Xiong, Cheng Shen, Jian Lin

**Affiliations:** 1 Department of Urology, Peking University First Hospital, No. 8 Xishiku Street, Xicheng District, Beijing 100034, China; 2 Institute of Urology, Peking University, Beijing 100034, China; 3 Beijing Key Laboratory of Precision Medicine and Innovative Translation for Urogenital Diseases, Beijing 100034, China; 4 National Urological Cancer Center, Beijing 100034, China

**Keywords:** Azvudine, COVID-19, kidney transplantation, long-term outcomes, renal function, immunosuppression

## Abstract

**Objectives**: Coronavirus disease 2019 (COVID-19) poses a significant threat to kidney transplant recipients (KTRs) due to chronic immunosuppression. While Azvudine has demonstrated antiviral efficacy, its long-term impact on allograft function in KTRs remains unknown. This single-arm retrospective study provides a comprehensive evaluation of the outcomes. **Methods**: This single-center, retrospective study consecutively enrolled 20 KTRs diagnosed with COVID-19 during the Omicron surge (December 2022–January 2023), all treated with an Azvudine-centered regimen. Clinical data, treatment responses, and serial renal function parameters were analyzed. Long-term follow-up extended to a median of 39 months. **Results**: The median time to COVID-19 nucleic acid negativity was 13.22 days. Renal function improved significantly during treatment: estimated glomerular filtration rate increased by a mean of 27.29 mL/min/1.73 m^2^, and serum creatinine decreased by 57.72 µmol/L (both *p* < 0.01). Common complications included electrolyte imbalances and co-infections (50% of patients). Crucially, at a median follow-up of 39 months, 75% (15/20) of patients maintained normal allograft function. No Azvudine-related adverse events or drug interactions with immunosuppressants were observed. **Conclusions**: While acknowledging the inherent limitations of a non-comparative design, this study provides preliminary evidence suggesting that an Azvudine-centered regimen may be associated with promising efficacy, a favorable safety profile without interference with immunosuppressive therapy, and—most importantly—durable renal allograft survival in KTRs with COVID-19. Our data suggest that this regimen could contribute to mitigating the long-term risk of allograft dysfunction, potentially bringing outcomes closer to the expected baseline for stable recipients. Coupled with its low risk of drug–drug interactions, potential immunomodulatory benefits, and superior cost-effectiveness, Azvudine could be considered a valuable therapeutic option for this high-risk population in the post-pandemic era, although these findings warrant cautious interpretation. Controlled trials are ultimately needed to confirm these observations.

## 1. Introduction

Coronavirus disease 2019 (COVID-19) continues to pose a significant threat to global health, particularly for immunocompromised individuals [1]. Among them, kidney transplant recipients (KTRs) represent a uniquely vulnerable population due to their chronic immunosuppressive state and frequent comorbidities, such as cardiovascular disease and chronic kidney disease. A study of transplant recipients across seven European countries reported an attributable mortality rate of 19.9% in KTRs diagnosed with COVID-19, which was markedly higher than that of the control group [2]. Another multicenter study involving 12 centers in the United States, Italy, and Spain further confirmed this elevated risk, with an initial mortality rate of 32% among KTRs [3].

The management of COVID-19 in KTRs is further complicated by potential drug–drug interactions between antiviral agents and critical immunosuppressants, such as calcineurin inhibitors. 2′-deoxy-2′-β-fluoro-4′-azidocytidine (Azvudine, FNC) is a novel nucleoside reverse transcriptase inhibitor originally developed for the treatment of HIV infection, originally developed in China and manufactured by Henan Zhenshi Bio-Technology Co., Ltd. (Pingdingshan, China) [4]. Azvudine inhibits severe acute respiratory syndrome coronavirus 2 (SARS-CoV-2) replication by terminating viral RNA/DNA strand synthesis [5]. Phase III trials and a recent meta-analysis have demonstrated its efficacy in reducing viral load, shortening time to clinical improvement, and lowering mortality in the general population. Its safety profile is comparable or superior to that of other agents, such as Paxlovid, particularly with regard to liver injury and intensive care unit (ICU) admission rates [6,7]. Promising results have also been observed in special populations, including the elderly and patients with cardiovascular diseases or malignancies [8,9,10].

However, despite its favorable pharmacokinetic profile—which suggests a low risk of interaction with immunosuppressants—robust clinical data on the use of Azvudine in solid organ transplant recipients are strikingly absent. Crucially, no study has yet reported its long-term impact on allograft function, a paramount concern in transplant care. This significant gap in knowledge limits evidence-based therapeutic choices for this high-risk group.

To address this gap, while acknowledging the constraints of a real-world observational study, we report clinical outcomes with an extended median follow-up of 39 months. We contextualize our findings by comparing them against previously published historical benchmarks for KTRs with COVID-19, providing critical context and offering a first transparent assessment of Azvudine’s real-world effectiveness.

## 2. Materials and Methods

Following the adjustment of China’s COVID-19 prevention policies in December 2022, a significant surge of Omicron variant infections occurred nationwide [11]. In this single-center, retrospective study, we consecutively included 20 KTRs who were admitted to Peking University First Hospital with COVID-19 pneumonia and received Azvudine treatment between December 2022 and January 2023.

### 2.1. Study Population and Design

The inclusion criteria were (a) diagnosis of COVID-19 confirmed by a positive polymerase chain reaction (PCR) test; (b) presence of moderate to severe clinical symptoms (e.g., fever, cough, dyspnea); (c) chest computed tomography (CT) findings consistent with viral pneumonia; and (d) maintenance on a long-term immunosuppressive regimen prior to admission. Patients were excluded if they were aged <18 years, had received other antiviral drugs (e.g., Paxlovid), or had a prior documented COVID-19 infection. All eligible patients during the study period were included; their enrollment timeline is illustrated in Supplementary Figure S1. Severity was assessed on admission using the World Health Organization (WHO) guideline [12], with severe disease defined as oxygen saturation < 90% on room air, signs of pneumonia, or signs of severe respiratory distress, and critical disease defined as requiring life-sustaining treatment, acute respiratory distress syndrome, sepsis, or septic shock. In this cohort, the mean age was 50.05 years (standard deviation [SD]: 11.45, range: 30–73), with 10 males and 10 females. Notably, 18/20 patients (90%) presented with severe disease and 2/20 (10%) with critical disease.

This is a single-arm, retrospective, observational study. We acknowledge that the lack of a concurrent control group represents a major limitation, dictated by the real-world urgency of the Omicron surge, during which withholding potentially beneficial antiviral therapy from any patient was deemed unethical. Consequently, our findings should be interpreted within the context of a prospective case series with rigorous longitudinal follow-up. A preprint has previously been published [13].

### 2.2. Treatment Protocol

**Figure 1:**
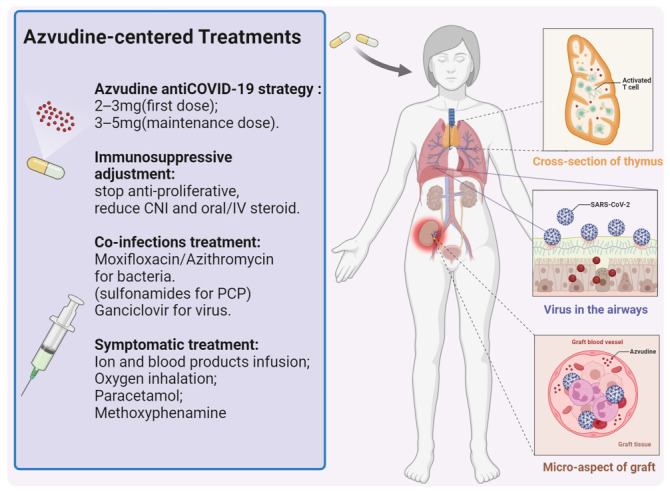
Introduction of Azvudine-centered treatment and possible mechanisms of Azvudine. PCP, Pneumocystis pneumonia.

All patients received a comprehensive Azvudine-centered treatment regimen; the key components are summarized in Figure 1. All initial management was conducted in the general ward. In accordance with institutional protocol, patients who developed respiratory failure would have been promptly transferred to the intensive care unit; however, no patient in this study required such a transfer.

#### 2.2.1. Azvudine Anti-COVID-19 Strategy

Oral Azvudine was initiated upon admission. The dosing regimen consisted of a first dose of 2–3 mg, followed by a maintenance dose of 3–5 mg daily. Treatment continued for 7–14 days until a negative SARS-CoV-2 nucleic acid test was achieved. The medication was administered at bedtime to improve tolerability.

#### 2.2.2. Immunosuppressive Adjustment

The concomitant immunosuppressive regimen was adjusted as follows: anti-proliferative agents (e.g., mycophenolate mofetil) were stopped; doses of calcineurin inhibitors (CNIs: tacrolimus or cyclosporine) were reduced to maintain low target trough levels; and oral or intravenous steroids were administered or increased to counteract inflammation.

#### 2.2.3. Co-Infections Treatment

For suspected or confirmed bacterial co-infections, moxifloxacin or azithromycin was used. For Pneumocystis pneumonia (PCP), sulfonamides were added. Ganciclovir was administered for cytomegalovirus (CMV) co-infection.

#### 2.2.4. Supportive and Symptomatic Treatment

Supportive care included infusion of ions and blood products to correct electrolyte imbalances and nutritional deficits; oxygen inhalation to maintain oxygen saturation (SpO_2_) ≥ 95%; paracetamol for fever; and methoxyphenamine for cough relief. Transplanted kidney function was closely monitored, and hemodialysis was initiated if necessary.

### 2.3. Data Collection and Outcomes

Demographic, clinical, laboratory, and treatment data were retrospectively collected from medical records. The primary outcomes included the time to SARS-CoV-2 nucleic acid conversion and changes in renal function. Renal allograft function was assessed using the following established parameters: (1) serum creatinine (SCr), a standard biomarker of glomerular filtration; (2) estimated glomerular filtration rate (eGFR), calculated using the Chronic Kidney Disease Epidemiology Collaboration (CKD-EPI) equation, which is validated for monitoring kidney function in transplant recipients [14]. These parameters were chosen because they are the most widely used and clinically validated measures of allograft function, recommended by the Kidney Disease: Improving Global Outcomes (KDIGO) Clinical Practice Guideline for the Care of Kidney Transplant Recipients [15]. Long-term renal outcomes were assessed during follow-up, extending to a median of 39 months. Safety was evaluated by monitoring for adverse events.

### 2.4. Statistical Analysis and Ethics

Continuous variables are presented as median (range) or mean ± standard deviation, as appropriate. Categorical variables are presented as numbers (percentages). Prior to analysis, the Shapiro–Wilk normality test was performed on the paired differences of all continuous parameters. For normally distributed differences (eGFR: W = 0.94, *p* > 0.05), the paired *t*-test was applied; for non-normally distributed differences (SCr: W = 0.73, *p* < 0.01), the Wilcoxon signed-rank test was used. A sensitivity analysis using the Wilcoxon test for eGFR yielded consistent results (*p* < 0.01). To evaluate the durability of renal function preservation, a Kaplan–Meier curve was constructed for the time to loss of renal function compliance (defined as serum creatinine ≤ 141 µmol/L) during the 1-year follow-up. Statistical significance was set at a two-sided *p*-value < 0.05. This study was approved by the Biomedical Research Ethics Committee of Peking University First Hospital (2023Research 244-001) and followed the World Medical Association Declaration of Helsinki as revised in 2024 [16]. The kidneys transplanted in the 20 patients in this study were obtained from donors who died in the ICU, in accordance with the principles of the Declaration of Istanbul [17,18].

## 3. Results

### 3.1. Patient Characteristics and Clinical Presentation

The baseline characteristics of the 20 included KTRs are summarized in Table 1. The most common maintenance immunosuppressive regimen was a combination of tacrolimus, mycophenolate mofetil, and prednisone (16 patients, 80%). Hypertension was the most prevalent comorbidity (9 patients, 45%), followed by diabetes mellitus (4 patients, 20%).

Following COVID-19 diagnosis, the most frequent symptom was high fever (18 patients, 90%), followed by production of white sputum (13 patients, 65%) and fatigue (10 patients, 50%). The median duration of Azvudine-centered therapy was 9.6 days (range: 7–14 days). The median time from treatment initiation to SARS-CoV-2 nucleic acid negativity was 13.22 days (range: 5–33 days). Notably, no patient required transfer to the intensive care unit.

### 3.2. Radiological Findings

**Figure 2:**
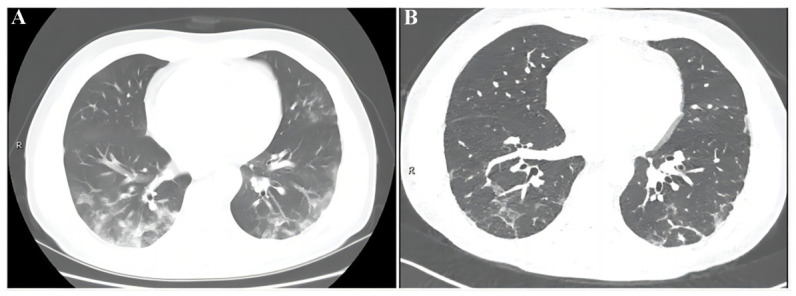
Comparison of CT imaging of the lungs before and after treatment: (**A**) pre-treatment shows multiple ground-glass opacities and consolidations (white areas) in the subpleural regions of the lower lobes; (**B**) post-treatment demonstrates marked reduction of these white areas.

Chest CT at admission typically revealed multiple ground-glass opacities and consolidations with scattered solid micronodules in both lungs, predominantly in the subpleural regions of the lower lobes (Figure 2A). These lesions were accompanied by bronchial wall thickening but were largely devoid of pleural effusion or cavitation. Following treatment, a significant reduction or complete resolution of these pulmonary lesions was observed in all patients (Figure 2B).

### 3.3. Laboratory Parameters and Renal Function Evolution

**Figure 3:**
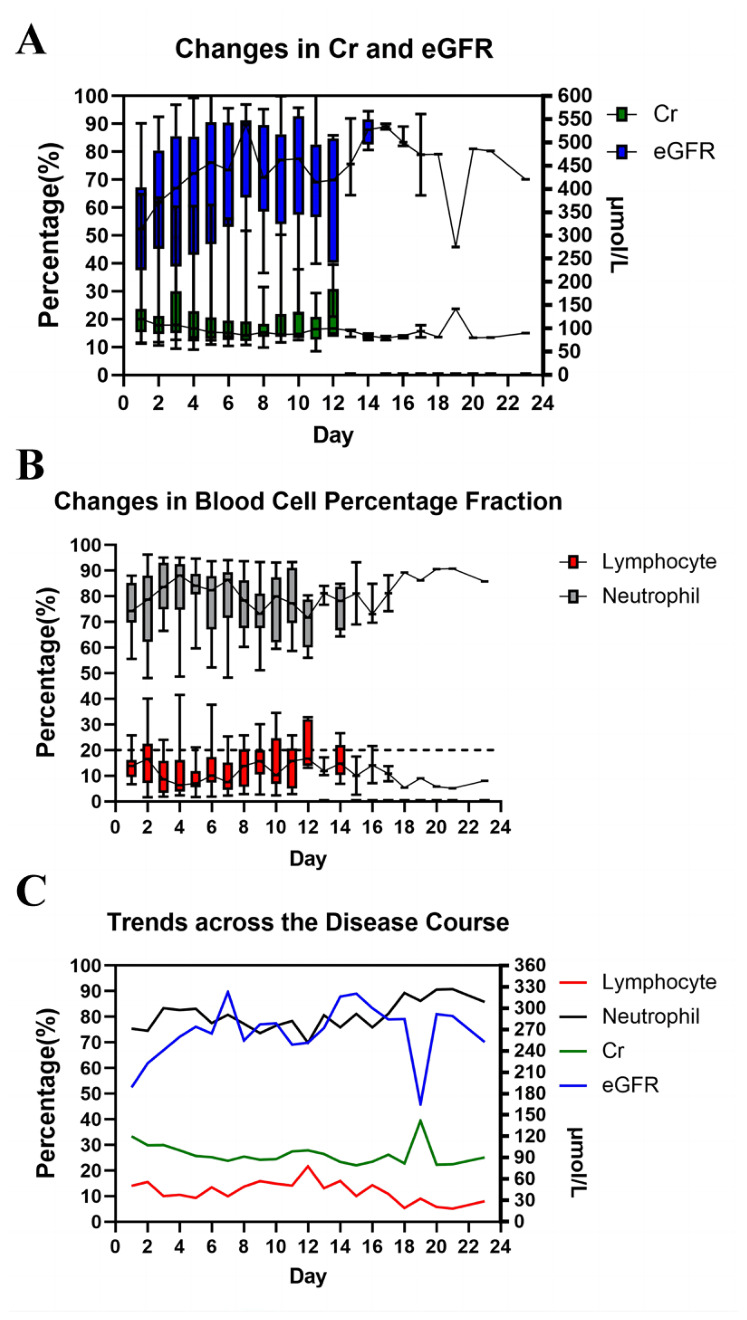
Changes in significant laboratory parameters during the course of the disease: (**A**) changes in serum Cr and eGFR; (**B**) changes in blood cell percentage fraction; (**C**) the overall trend of the disease course. Data in (**A**,**B**) are presented as box plots with individual data points, demonstrating data distribution and central tendency. (**C**) Multi-parameter trends across all measured days. To avoid visual confusion, only mean values are plotted. The majority of patients (80%, 16/20) achieved clinical and virological recovery within 14 days; data points beyond Day 14 reflect the remaining 4 patients, with only 2 patients remaining after Day 18–20. Transient fluctuations in eGFR and Cr after Day 14 were primarily attributable to concurrent infections other than COVID-19 and resolved after comprehensive treatment. Cr, creatinine; eGFR, estimated glomerular filtration rate.

Serial laboratory findings were analyzed. Figure 3 illustrates the dynamic changes in SCr and eGFR throughout the disease course.

The mean eGFR at admission was 54.13 (SD: 22.41) mL/min/1.73 m^2^, with 5 patients (25%) having a serum creatinine > 141 μmol/L, indicative of pre-existing allograft dysfunction exacerbated by acute infection. Following Azvudine administration, a continuous decline in SCr was observed. Notably, the improvement in renal function was not random. A within-subject, before-and-after analysis revealed a consistent and rapid upward trend in eGFR in 14 out of 20 patients (70%) within the first three days of Azvudine therapy, illustrating a clear temporal relationship with treatment initiation. After 10 days of treatment, patients exhibited a significant improvement in renal function compared to baseline: the mean eGFR increased by 21.39 (SD: 12.65) mL/min/1.73 m^2^ (t = 7.56, *p* < 0.01, 95% confidence interval [CI]: [15.47, 27.31]), and the mean SCr decreased by 35.04 (interquartile range [IQR]: 26.265) μmol/L (z = 3.92, *p* < 0.01). Moreover, the majority of patients (16/20, 80%) achieved clinical and virological recovery within 14 days of treatment, with stable or improving eGFR and serum Cr levels (Figure 3C). After Day 14, the analysis included the remaining 4 patients (with only 2 patients after Days 18–20) who experienced broader clinical complications. Transient decreases in eGFR and increases in serum Cr were observed in these patients during Days 14–20, largely attributable to concurrent infections other than COVID-19. These parameters subsequently improved following comprehensive supportive care, indicating that the fluctuations were transient and not causally related to Azvudine therapy.

Hematological analysis revealed that lymphopenia was common during active infection, with 10 patients (50%) showing a lymphocyte percentage < 20%. This trend reversed with clinical improvement. Furthermore, electrolyte disturbances were highly prevalent: hypomagnesemia (19 patients, 95%), hypocalcemia (18 patients, 90%), hyponatremia (10 patients, 50%), and hypokalemia (5 patients, 25%).

### 3.4. Co-Infections

The most common were Mycoplasma pneumoniae (7 patients), followed by JC virus and Legionella pneumophila (5 patients each), and cytomegalovirus (4 patients).

### 3.5. Treatment Cost

The average cumulative dose of Azvudine per patient was 37.3 mg (range: 16–58 mg). The corresponding mean drug cost was 220.44 Chinese Yuan (RMB), with detailed costs for each patient provided in Supplementary Table S1.

### 3.6. Long-Term Renal Outcomes and Safety

Long-term renal allograft function was the paramount outcome of this study. At the 1-year follow-up, renal function remained stable in most patients compared to their post-treatment levels (Supplementary Figure S2). Fourteen patients (70%) maintained normal graft function (SCr ≤ 141 μmol/L), while six had impaired function. No significant differences in age, medication duration, or viral clearance time were found between these two groups (Supplementary Table S2).

Crucially, with a median follow-up extended to 39 months (interquartile range [IQR]: 38, 39.5) as of May 2026, the durability of this outcome was confirmed. At this long-term endpoint, 15 out of 20 patients (75%) maintained serum creatinine within the normal range. One patient experienced graft loss due to unrelated causes, and four developed renal insufficiency. Throughout the entire follow-up period, no patient required re-admission for COVID-19 reinfection, and no Azvudine-related adverse events or complications were observed.

## 4. Discussion

This single-center retrospective study provides the first clinical evidence on the efficacy, safety, and—crucially—long-term renal outcomes of an Azvudine-centered regimen for treating COVID-19 in KTRs. Our findings, supported by a median follow-up of 39 months, suggest that Azvudine is a viable and promising therapeutic option for this high-risk immunocompromised population.

### 4.1. Confirming Efficacy in an Immunosuppressed Setting

Prior studies have established Azvudine’s efficacy in shortening viral clearance time and reducing disease progression in the general population [5,19,20]. The pivotal question addressed here is its effectiveness under chronic immunosuppression. Our results affirm its utility in KTRs: all patients achieved symptomatic relief, with a median time to nucleic acid negativity of 13.22 days. While this conversion time is longer than that reported in immunocompetent cohorts, it is clinically reasonable and likely reflects the attenuated immune response inherent to KTRs. Importantly, no patient required intensive care unit admission, indicating the regimen’s effectiveness in preventing critical disease progression.

### 4.2. Preservation of Renal Allograft Function

The paramount concern in treating KTRs is the preservation of long-term allograft function, and our study is the first to report on this critical endpoint. To interpret our findings meaningfully, we compared the observed long-term outcomes to the expected natural history of KTRs. According to Hariharan et al. [21], 3-year graft survival rates across 15 countries ranged from 80% to 95% for deceased donor transplants and from 88% to 97% for living donor transplants. In our cohort, with a median follow-up of 39 months, 75% of patients (15/20) maintained normal serum creatinine, and critically, only one patient (5%) experienced graft loss due to non-adherence rather than COVID-19 or its treatment. While the sample size is modest, this contextual comparison suggests that the Azvudine-centered regimen may have helped preserve allograft function at a level closely approaching the expected baseline for stable KTRs without a COVID-19 event.

This favorable renal profile may be partly attributed to Azvudine’s minimal risk of clinically significant drug–drug interactions with maintenance immunosuppressants. This stands in contrast to agents like Paxlovid (nirmatrelvir/ritonavir), where the ritonavir component is a potent inhibitor of cytochrome P450 3A4 and P-glycoprotein, posing a high risk of elevating calcineurin inhibitor levels and subsequent toxicity [22,23]. Azvudine’s distinct nucleoside analog mechanism avoids these pharmacokinetic interactions, thereby simplifying clinical management and enhancing its safety profile in transplant recipients.

Acknowledging the absence of a control group, we contextualized our findings against a recently published historical benchmark from the same COVID-19 period in China. A single-center cohort study of 324 hospitalized KTRs with COVID-19 during the Omicron wave (December 2022–January 2023) reported a 13.0% overall mortality rate, a 12.0% ICU admission rate, a 25.0% acute kidney injury (AKI) incidence, and a 15.4% graft loss rate [24]. Furthermore, the authors noted that the use of immunomodulators and late antiviral therapy did not improve survival. In striking contrast, our cohort of 20 KTRs treated with an early Azvudine-centered regimen exhibited zero mortality, zero ICU admission, zero incidence of AKI requiring dialysis, and only 5% (1 patient) graft loss from an unrelated cause at a median follow-up of 39 months. Moreover, 75% of our patients maintained normal allograft function long-term. While acknowledging the small sample size of our study, this direct comparison to a large, same-period cohort suggests that an early, focused Azvudine regimen may effectively mitigate the significant risks of mortality, severe disease, and long-term graft dysfunction that are observed in this vulnerable population during the Omicron surge.

We acknowledge that the observed clinical improvement cannot be solely attributed to Azvudine, given the concurrent immunosuppressive adjustments, anti-infective therapies, and fluid/electrolyte management—all standard treatments for managing the acute phase of COVID-19. However, we propose that Azvudine played a pivotal role for several reasons. First, from a causal mechanism perspective, the rapid reversal of lymphopenia and decline in serum creatinine, coinciding with viral clearance, aligns with Azvudine’s known antiviral mechanism rather than being a direct effect of immunosuppressive reduction or antibiotics. Second, our cohort’s zero incidence of AKI requiring dialysis stands in marked contrast to the 25% AKI rate and 10.8% RRT rate reported in the contemporaneous cohort by Lv et al. [24], strongly suggesting a specific renoprotective effect of early Azvudine therapy. Third, concerning confounding, while immune management (e.g., reducing mycophenolate mofetil) can theoretically mitigate inflammatory damage, such changes are typically slow and are contraindicated during active infection. The swift (within days) and consistent renal improvement we observed is more congruent with a direct antiviral effect mitigating viral-induced tubular injury and the cytokine storm.

Based on this single-arm study, we cannot conclude that Azvudine completely eliminated the excess risk of allograft dysfunction from COVID-19. However, our findings strongly suggest that the risk was effectively mitigated. With no COVID-19-related re-admissions and only one case of graft loss from an unrelated cause, our data provide compelling evidence that the Azvudine regimen protected against long-term, COVID-19-specific graft damage, bringing outcomes close to the expected baseline for stable recipients. Controlled studies are needed to precisely quantify the risk reduction.

### 4.3. Potential Immunomodulatory Mechanisms

Beyond its direct antiviral action, Azvudine may offer beneficial immunomodulatory effects. Its active form concentrates in the thymus [25], potentially regulating cytokine expression by promoting anti-inflammatory interleukins (IL-4, IL-10, and IL-13) and suppressing pro-inflammatory cytokines (IL-1β, interferon-γ [IFN-γ], tumor necrosis factor-α [TNF-α], and IL-6) that are involved in the COVID-19-associated cytokine storm. This immunomodulatory capacity is consistent with our observed reversal of lymphopenia following treatment, a hematological trend also noted in other studies [26]. Monitoring lymphocyte dynamics may therefore serve as a useful biomarker for tracking disease course in KTRs with COVID-19.

### 4.4. Clinical Insights from Prevalent Electrolyte Imbalances

We documented a high prevalence of electrolyte disorders (e.g., hypomagnesemia in 95% of patients and hypocalcemia in 90%). These imbalances may reflect both the systemic inflammatory state of COVID-19 and potential subclinical renal tubular injury, despite the observed improvement in glomerular filtration markers. This finding underscores the necessity for vigilant monitoring and proactive correction of electrolytes in KTRs with COVID-19, which is critical not only for renal health but also for the maintenance of cardiac function.

### 4.5. Cost-Effectiveness and Implications for Accessibility

The economic aspect of treatment is a practical consideration. In our cohort, the average drug cost for a full Azvudine course was 220.44 RMB, which is substantially lower than the approximate 1800 RMB cost for a Paxlovid course [27]. This significant cost advantage enhances the accessibility of effective antiviral therapy, particularly in resource-limited settings or for patients with financial constraints.

### 4.6. Limitations and Future Directions

Our study has several limitations that should be acknowledged. First, the retrospective, single-arm design without a concurrent control group precludes definitive causal inference. The lack of a control cohort was a consequence of the real-world urgency of the pandemic, during which a placebo-controlled design was not feasible. Second, the treatment regimen was multimodal, including immunosuppressive adjustment, anti-infective therapy, and supportive care. While we have provided logical arguments and historical comparisons to isolate Azvudine’s effect, it remains impossible to fully disentangle its contribution from that of co-interventions. Third, while a strength, our long-term follow-up (median 39 months) lacks a matched control group, preventing us from concluding that Azvudine completely eliminates the COVID-19-associated excess risk of allograft dysfunction. Finally, the modest sample size (*n* = 20), though understandable for this specialized patient group, warrants validation in larger, multi-center studies. Future prospective randomized controlled trials are essential to confirm our findings and definitively establish the role of Azvudine in managing COVID-19 in solid organ transplant recipients.

## 5. Conclusions

In conclusion, this single-arm observational study provides preliminary evidence suggesting that an Azvudine-centered regimen may be associated with promising efficacy, a favorable safety profile, and—most importantly—durable renal allograft survival in KTRs with COVID-19. Our data suggest that this regimen could contribute to mitigating the long-term risk of allograft dysfunction, potentially bringing outcomes closer to the expected baseline for stable recipients. Coupled with its low risk of drug–drug interactions, potential immunomodulatory benefits, and superior cost-effectiveness, Azvudine could be considered a valuable therapeutic option for this high-risk population in the post-pandemic era, although these findings warrant cautious interpretation. Controlled trials are ultimately needed to confirm these observations.

## Figures and Tables

**Table 1:** The patients’ characteristics.

Characteristics	Results
Total number of patients, *n*	20
Gender, *n* (%)	
Male	10 (50%)
Female	10 (50%)
Age (yr), mean (SD)	50.5 (11.45)
Comorbidity, *n* (%)	
Hypertension	9 (45%)
Hyperglycemia	4 (20%)
Cardiopathy	2 (10%)
Hepatitis B	1 (5%)
Time after transplantation, *n* (%)	
<1 year	2 (10%)
1–10 years	6 (30%)
>10 years	12 (60%)
Medication duration (day), median (range)	9.6 (7–14)
Immunosuppressive schemes, *n* (%)	
Tacrolimus + MMF + Prednisone	16 (80%)
Cyclosporin A	4 (20%)
Symptoms, *n* (%)	
High fever	18 (90%)
Fatigue	10 (50%)
White sputum	13 (65%)
Yellow sputum	5 (25%)
Chest tightness	3 (15%)
Hyposmia	2 (10%)
Hypogeusia	1 (5%)
Nausea	2 (10%)
Severity, *n* (%)	
Severe	18 (90%)
Critical	2 (10%)
Baseline serum Cr (μmol/L), median (IQR)	118.00 (94.53, 141.3)
Baseline eGFR (mL/min/1.73 m^2^), mean (SD)	54.13 (22.41)
Conversion time (day), median (range)	13.22 (5–33)

MMF, mycophenolate mofetil; Cr, creatinine; eGFR, estimated glomerular filtration rate; IQR, interquartile range; SD, standard deviation.

## Data Availability

The datasets used and analyzed during the current study are available from the corresponding author upon reasonable request.

## Supplementary Materials

The following supporting information can be downloaded at https://www.mdpi.com/article/10.3390/jcm15145417/s1, Figure S1: Inclusion of patients in the study; Figure S2: Changes in renal function during the 1-year post-treatment period in patients (compliance rate of serum creatinine means the rate of patients with blood creatinine ≤ 141 µmol/L); Table S1: Azvudine dose and cost of each patient; Table S2: Comparison between patients with serum creatinine > 141 µmol/L and ≤141 µmol/L.

## Abbreviations

The following abbreviations are used in this manuscript:

COVID-19	coronavirus disease 2019
KTRs	kidney transplant recipients
FNC	Azvudine
SARS-CoV-2	severe acute respiratory syndrome coronavirus 2
ICU	intensive care unit
PCR	polymerase chain reaction
CT	computed tomography
WHO	World Health Organization
SD	standard deviation
CNI	calcineurin inhibitor
PCP	pneumocystis pneumonia
CMV	cytomegalovirus
SpO_2_	oxygen saturation
SCr	serum creatinine
Cr	creatinine
eGFR	estimated glomerular filtration rate
CKD-EPI	Chronic Kidney Disease Epidemiology Collaboration
KDIGO	Kidney Disease: Improving Global Outcomes
MMF	mycophenolate mofetil
CI	confidence interval
RMB	Renminbi
IQR	interquartile range
IL	interleukin
INF	interferon
TNF	tumor necrosis factor
AKI	acute kidney injury
