# Supplementary material for: Azvudine for COVID-19 in Kidney Transplant Recipients: A Real-World Observational Study of Long-Term Renal Outcomes
Source: J Clin Med. 2026 Jul 10;15(14):5417. doi: 10.3390/jcm15145417 (PMC13412598; doi:10.3390/jcm15145417)
Supplement: Supplementary file 1 [file jcm-15-05417-s001.zip › jcm-4363577-supplementary.pdf]

Supplementary Information

Supplementary material included Supplementary Figure S1, Supplementary Figure S2, Supplementary Table S1 and Supplementary Table S2.

Supplementary Figure S1. Inclusion of patients in the study.

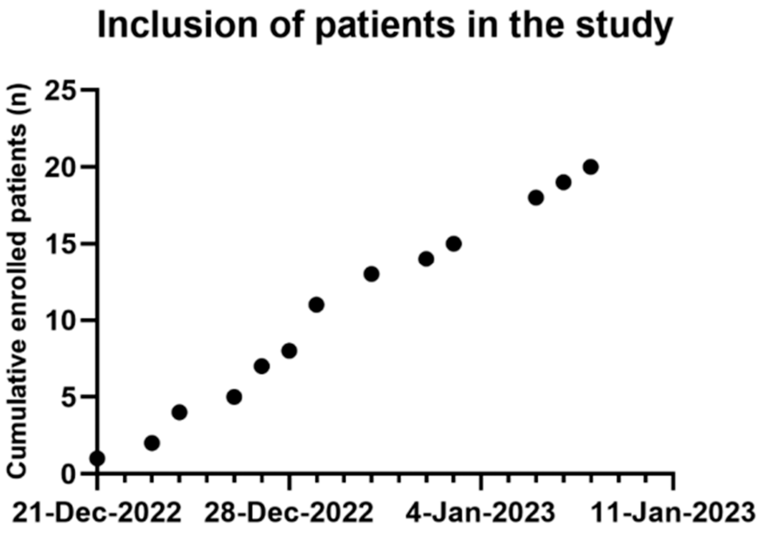

Supplementary Figure S2. Changes in renal function during the 1-year post-treatment period in patients (Compliance rate of serum creatinine means the rate of patients with blood creatinine ≤ 141 μmol/L).

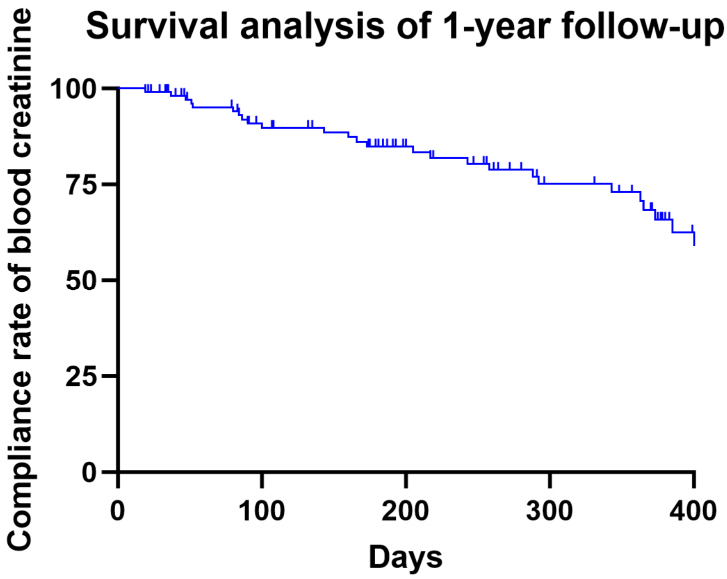

Supplementary Table S1. Azvudine dose and cost of each patient.

Patient's number	Dose of Azvudine (mg)	Cost of Azvudine (RMB)
1	41	242.31
2	27	159.57
3	38	224.58
4	35	206.85
5	35	206.85
6	48	283.68
7	31	183.21
8	31	183.21
9	47	277.77
10	35	206.85
11	24	141.84
12	43	254.13
13	16	94.56
14	41	242.31
15	40	236.4
16	43	254.13
17	36	212.76
18	27	159.57
19	58	342.78
20	55	325.05

RMB, Renminbi.

Supplementary Table S2. Comparison between patients with serum creatinine > 141 μmol/L and ≤ 141 μmol/L.

Characteristics	Patients with blood creatinine > 141 μmol/L	Patients with blood creatinine ≤ 141 μmol/L	P-value
Gender,n(%)			/
Male	2（33）	8（57）	
Female	4（67）	6（43）	
Age(yr),median(range)	43（30-53）	53.5(37-73)	0.0528
Comorbidity,n(%)			/
Hypertension	2(100%)	7(50)	
Hyperglycemia	0	4(29)	
Cardiopathy	0	2(14)	
Hepatitis B	0	1(7)	
Medication duration(day),median(range)	9.5(8-11)	10(6-22)	0.8238
Immunosuppresive schemes,n(%)			/
FK+M+P	5(83)	11(79)	
Cyclosporin A	1(17)	3(21)	
Symptoms,n(%)			/
High fever	5(83)	13(93)	
Fatigue	5(83)	5(36)	
White sputum	3(50)	10(71)	
Yellow sputum	2(33)	3(21)	
Chest tightness	1(17)	2(14)	
Hyposmia	1(17)	1(7)	
Hypogeusia	0	1(7)	
Nausea	1(17)	1(7)	
Conversion time(day), median(range)	11(7-33)	12(5-25)	0.7197
